# Malaria, helminths, co-infection and anaemia in a cohort of children from Mutengene, south western Cameroon

**DOI:** 10.1186/s12936-016-1111-2

**Published:** 2016-02-06

**Authors:** Clarisse Njua-Yafi, Eric A. Achidi, Judith K. Anchang-Kimbi, Tobias O. Apinjoh, Regina N. Mugri, Hanesh F. Chi, Rolland B. Tata, Charles Njumkeng, Emmanuel N. Nkock, Theresa Nkuo-Akenji

**Affiliations:** Department of Animal Biology and Physiology, University of Yaounde I, Yaounde, Cameroon; Department of Microbiology and Parasitology, University of Buea, Buea, Cameroon; Department of Zoology and Animal Physiology, University of Buea, Buea, Cameroon; Department of Biochemistry and Molecular Biology, University of Buea, Buea, Cameroon; Department of Medical Laboratory Science, University of Buea, Buea, Cameroon

**Keywords:** Malaria, Helminths, Co-infection, Anaemia, Children

## Abstract

**Background:**

Malaria and helminthiases frequently co-infect the same individuals in endemic zones. *Plasmodium falciparum* and helminth infections have long been recognized as major contributors to anaemia in endemic countries. Several studies have explored the influence of helminth infections on the course of malaria in humans but how these parasites interact within co-infected individuals remains controversial.

**Methods:**

In a community-based longitudinal study from March 2011 to February 2012, the clinical and malaria parasitaemia status of a cohort of 357 children aged 6 months to 10 years living in Mutengene, south-western region of Cameroon, was monitored. Following the determination of baseline malaria/helminths status and haemoglobin levels, the incidence of malaria and anaemia status was determined in a 12 months longitudinal study by both active and passive case detection.

**Results:**

Among all the children who completed the study, 32.5 % (116/357) of them had at least one malaria episode. The mean (±SEM) number of malaria attacks per year was 1.44 ± 0.062 (range: 1–4 episodes) with the highest incidence of episodes occuring during the rainy season months of March–October. Children <5 years old were exposed to more malaria attacks [OR = 2.34, 95 % CI (1.15–4.75), p = 0.019] and were also more susceptible to anaemia [OR = 2.24, 95 % CI (1.85–4.23), p = 0.013] compared to older children (5–10 years old). Likewise children with malaria episodes [OR = 4.45, 95 % CI (1.66–11.94), p = 0.003] as well as those with asymptomatic parasitaemia [OR = 2.41, 95 % CI (1.58–3.69) p < 0.001] were susceptible to anaemia compared to their malaria parasitaemia negative counterparts. Considering children infected with *Plasmodium* alone as the reference, children infected with helminths alone were associated with protection from anaemia [OR = 0.357, 95 % CI (0.141–0.901), p = 0.029]. The mean haemoglobin level (g/dl) of participants co-infected with *Plasmodium* and helminths was higher (p = 0.006) compared to participants infected with *Plasmodium* or helminths alone.

**Conclusion:**

Children below 5 years of age were more susceptible to malaria and anaemia. The high prevalence of anaemia in this community was largely due to malaria parasitaemia. Malaria and helminths co-infection was protective against anaemia.

**Electronic supplementary material:**

The online version of this article (doi:10.1186/s12936-016-1111-2) contains supplementary material, which is available to authorized users.

## Background

Malaria and helminthiases, two of the most prevalent infections affecting humans, overlap extensively in their epidemiological distributions and frequently co-infect in the same individuals [1–9]. Human intestinal helminthiasis is most commonly caused by soil-transmitted helminths (STHs), namely *Ascaris lumbricoides*, *Trichuris trichiura*, and the hookworms *Necator americanus* and *Ancylostoma duodenale* [3, 4, 8]. Although the levels of helminth infections are generally low in young children, resulting in few *Plasmodium*–helminth co-infections [10], school-aged children, rather than pre-school children or adults, are at greatest risk of co-infections and its consequences [2, 3].

Various studies have explored the influence of helminth infections on the course of malaria in humans but whether and how these two parasites interact within co-infected hosts remains controversial [1, 2, 4, 7, 11, 12]. Co-infecting parasites may interact either positively or negatively in the receptive hosts [6, 10, 13] through a range of mechanisms including resource competition, immune-mediated interactions and direct interference [7, 8]. An earlier study performed in 1978 reported that anti-helminthic treatment of severe ascariasis in a high-transmission area was followed by an increase in symptomatic malaria suggesting a protective role of helminths against malaria. However, several studies have shown that infection with helminths increases susceptibility to malaria infection [1, 2, 9]. Recent studies have shown a positive association between hookworm infection and asymptomatic *Plasmodium falciparum* parasitaemia [8], while individuals with light *Trichuris trichiura* and *Ascaris lumbricoides* infections had lower *Plasmodium* densities than those with moderate or heavy infection [9]. Nevertheless, no association was found between STH infection and malaria in Uganda [2].

Although the aetiology of anaemia is complex, *P. falciparum* and helminth infections have long been recognized as major contributors to anaemia in endemic countries. Malarial anaemia is more typically associated with the acute clinical state, but there is evidence to suggest that asymptomatic malaria may contribute substantially to anaemia in endemic regions [10] through mechanisms such as haemolysis, increased spleen clearance of infected and uninfected red blood cells and cytokine induced dyserythropoesis. Intestinal helminths may cause anaemia as a result of direct blood loss, nutritional theft and impairment of appetite due to immunological factors [6, 8, 10, 13]. There is evidence that *Ascaris lumbricoides*-associated vitamin A deficiency may further increase the risk of anaemia in those co-infected with malaria [10]. Previous studies have shown that malaria–helminth co-infected individuals had significantly higher prevalence of anaemia and lower mean haemoglobin concentration compared to those infected with malaria only [3, 6, 12]. In the South West Region of Cameroon, school children infected exclusively with *P. falciparum* had higher levels of anaemia compared with children with co-infections, helminth infection only or uninfected [11].

The association between malaria susceptibility and helminth infection seems to be influenced by the type of helminth infection, the intensity of infection and the age of the population studied [5]. So far all the studies conducted on malaria–helminth co-infections in the South West Region are cross sectional. Longitudinal studies of malaria and helminth infections in well-defined cohorts will provide more valuable data on the epidemiology and/or control of these parasitic diseases. This study was, therefore, conducted to assess the effect of intestinal helminths infection on the prevalence and/or incidence of malaria and their combined impact on anaemia in children resident in Mutengene, South West Region of Cameroon.

## Methods

### Study area

The study was carried out in Mutengene, a semi-urban community located in the Mt Cameroon region, Fako division of the South West Region of Cameroon between February 2011 and March 2012. The study community comprised ten residential quarters spanning the entire Mutengene community. Volunteer study participants were sampled either at the Mutengene Medical Integrated Health Centre or at temporary sampling sites within their respective quarters in the community. The community has a Medical Integrated Health Centre which is the only government owned institution that offers affordable health services to the community. This town is located at about 220 m above sea level and has a heterogeneous population of approximately 40,000 inhabitants who originate mainly from neighbouring regions in search of its fertile farmland and business opportunities [14, 15]. The Mutengene health area has an equatorial climate that consists of a short dry season (late November to February) and a long rainy season (March–November) with maximum rainfall between June and August interspersed by early moderate (March–May) and late moderate (September–November) rains [14]. It is characterized by mean temperatures of 28 °C, 299.2 mm of rainfall and mean relative humidity of 87 % [16]. The Mount Cameroon region is hyperendemic for malaria [14, 17, 18], with the highest entomological inoculation rate (EIR) reaching an annual average of 1077.1 infectious bites/person/year at Esuke Camp in Mutengene [19]. Malaria transmission is more intense during the rainy season with peak transmission during the heavy rains. The main malaria vectors are *Anopheles gambiae, Anopheles funestus* and *Anopheles hancocki. Plasmodium falciparum,* the predominant malaria parasite species, accounts for up to 96 % of malaria infections in the study area [20].

### Study population and sampling procedure

This study was a community-based longitudinal study of a cohort of children aged between 6 months and 10 years from randomly selected households in the community. After randomly selecting 10 (50 %) of the 20 quarters in the study community, households within the selected quarters also went through systematic random selection. Approval for this study was obtained from the Ministry of Health and from the National Ethics Committee. Following informed consent from parents/guardians, study participants were recruited from households resident in the community. The incidence of malaria was determined by both passive case detection through self-referral to the Mutengene Health Centre (or other health establishments) and active case detection using a validated morbidity monitoring questionnaire (biweekly home visits and sampling every 3 months). During the home visits febrile children were brought to the health centre for clinical examination and treatment. Finger prick blood samples were collected from all febrile/sick children at the health centre for haemoglobin measurement and malaria parasitaemia detection. Anthropometric measurements and axillary temperature of participants were recorded every trimester from enrolment till 1 year and venous blood samples were collected at enrolment, at 6 months and at 12 months, while finger prick samples were collected at 3 and 9 months for malaria parasitaemia detection and haemoglobin measurement. An episode of malaria was defined as fever with an axillary temperature >37.5 °C, the presence of malaria parasites in a thick blood film and one other sign/symptom of malaria (shaking chills, headache, muscle ache, vomiting or fatique). Children presenting with acute malaria during the study period were given artemether–lumefantrine, a first-line ACT combination currently recommended for use in the treatment of uncomplicated malaria in Cameroon. Stool samples were also collected at enrolment and 6 months later for the detection of intestinal helminths. All participants had access to free consultation, malaria tests and treatment throughout the study period.

### Haematological analysis

Full blood count was determined in venous blood using an automated haemoanalyser (Teco, USA). Anaemia was defined as a haemoglobin (Hb) level <11 g/dl and was further classified based on WHO guidelines as severe anaemia: Hb <7 g/dl, moderate anaemia: Hb 7–9.9 g/dl, mild anaemia: Hb 10–10.9 g/dl [21].

### Malaria parasitaemia determination

Thick and thin blood films prepared from blood samples following standard procedures were stained with 10 % Giemsa (Sigma, St Louis, USA). Malaria parasitaemia status, density and species were determined by light microscopy using an Olympus microscope (Olympus Optical Co., Ltd, Japan). Smears were reported as negative only after observing at least 100 high power fields. Using the white blood cell (WBC) count per μl of blood of each participant, the parasitaemia density/μl was estimated by counting the number of asexual parasites against a minimum of 200 leukocytes and calculated using the following formula:

Parasitaemia density (parasites/µl of blood) = (No of parasites × WBC count/µl)/(No of WBCs counted).

### Intestinal helminths determination

Intestinal helminths were detected in stool samples using the formol-ether sedimentation concentration technique as described by Ash and Orihel [22]. Approximately 1 g of stool was washed using 10 % formol-ether solutions and the pellet stained with a drop of Lugol’s iodine. The ova or larvae of helminths were identified by light microscopy (Olympus Optical Co., Ltd, Japan) and the infection quantified as the mean egg per gram (epg) of stool.

### Statistical analysis

Data was entered into Excel and analysed using SPSS Statistics version 20 for windows (SPSS Inc, Chicago USA). Malaria parasitaemia and helminth densities were log-transformed before analysis. Differences in groups means were assessed using the one-way analyses of variance (ANOVA), while differences in proportions were assessed using Chi square. Associations between helminth infection, malaria–helminths co-infection and anaemia were assessed by binary logistic regression. The levels of correlation between variables were determined by calculating Pearson’s correlation coefficient (r). A difference or correlation giving a p value ≤0.05 was considered statistically significant.

## Results

### Baseline, 6 and 12 months characteristics of the study population

A total of 374 children aged 6 months to 10 years (mean age ± SEM: 4.39 ± 0.129 years) were enrolled from ten residential quarters of the Mutengene Health Area. At baseline 25.4, 18, 19.8 and 71.5 % of the participants were febrile, malaria parasitaemia (mp) positive, helminths positive and anaemic (severe: 2.6 %, moderate: 51.9 % and mild: 45.5 %) respectively. Among mp positive cases, 25.4, 73.1 and 18 % were febrile, anaemic and positive for helminths respectively (Table 1). At baseline and at 12 months, malaria parasitaemia was more prevalent (68.7 % and 72.2 % respectively) in the 5–10 years old age group (p ≤ 0.031) while at 6 months the prevalence of malaria parasitaemia was similar (p = 0.492) in both age groups. Malaria parasitaemia prevalence increased from 18 % at baseline to 19.3 % at 6 months and decreased to 12 % at 12 months. There was no difference in the proportion of febrile cases between mp positive and mp negative cases at baseline or at 6 months but at 12 months all febrile cases were mp positive. At 6 and 12 months, a higher proportion of mp positive participants were anaemic (p = 0.002 and p = 0.001, respectively) compared to mp negative participants but no such difference (p = 0.733) was observed at baseline. Participants were categorized into three groups: those without detectable malaria parasitaemia by microscopy and no clinical manifestation of malaria (P− M−), those who were asymptomatic but were positive for malaria parasitaemia (P+ M−) and those who were febrile and malaria parasitaemic plus at least one sign/symptom of malaria (P+ M+). Participants who were asymptomatic, but malaria parasitaemia positive (P+ M−) were significantly (p < 0.001) older compared to participants in the P+ M+ and P− M− groups. Similarly the haemoglobin levels of participants in the P− M− were significantly (p < 0.001) higher compared to those in the P+ M− and P+ M+ in decreasing order. The study also found a significantly (p = 0.012) higher parasitaemia density in the P+ M+ group compared to the P+ M− group (Table 1).

**Table 1:** Characteristics of malaria parasitaemia positive and negative children in a 1 year longitudinal cohort study

Parameter	Subclass	Malaria parasitaemia Status % (n)						p value
		Enrolment ^a^		6 months ^b^		12 months^c^		
		Pos	Neg	Pos	Neg	Pos	Neg	
Age group (years)	<5	31.3 (21)	58.4 (178)	42.6 (23)	47.8 (108)	27.8 (10)	52.1 (138)	0.001^a,^* 0.492^b^ 0.031^c,^*
	5–10	68.7 (46)	41.6 (127)	57.4 (31)	52.2 (118)	72.2 (26)	47.9 (127)	
	% (n)	18 (67)	82 (305)	19.3 (54)	80.7(226)	12 (36)	88 (265)	
Gender	Male	47.8 (32)	53.6 (164)	53.7 (29)	50.4 (114)	52.8 (19)	52.5 (139)	0.360^a^ 0.625^b^ 0.971^c^
	Female	52.2 (35)	46.4 (142)	46.3 (25)	49.6 (112)	47.2 (17)	47.5 (126)	
	n	67	306	54	226	36	265	
Anaemia status	Anaemic	73.1 (49)	71.1 (216)	79.2 (42)	55.7 (122)	69.4 (25)	40 (106)	0.733^a^ 0.002^b,^* 0.001^c,^*
	Non anaemic	26.9 (18)	28.9 (88)	20.8 (11)	44.3 (97)	30.6 (11)	60 (159)	
	n	67	304	53	219	36	265	
Febrile	Yes	25.4 (17)	20 (61)	16.7 (9)	8.4 (19)	8.3 (3)	0 (0)	0.328^a^ 0.069^b^ 0.001^c,^*
	No	74.6 (50)	80 (244)	83.3 (45)	91.6 (207)	91.7 (33)	100 (265)	
	n	67	305	54	226	36	265	
Helminth status	Positive	18 (11)	20.2 (52)	3.7 (1)	11.8 (13)	–	–	0.698^a^ 0.212^b^
	Negative	82 (50)	79.8 (205)	96.3 (26)	88.2 (97)	–	–	
	n	61	257	27	110	–	–	

	P+ M+ (%)	P+ M− (%)	P− M− (%)	
	Classification of participants (Mean ± SEM)			
Age (years)	5.18 ± 0.38 (2.9)	5.55 ± 0.20 (12.8)	4.65 ± 0.08 (84.3)	<0.001^§^
Haemoglobin (g/dl)	9.08 ± 0.34 (3.0)	9.93 ± 0.13 (12.9)	10.62 ± 0.05 (84.1)	<0.001^§^
Mp density (/µl)	3.32 ± 0.16 (18.5)	2.91 ± 0.07 (81.5)	–	0.012^§^

*Statistical significance determined by Chi square analysis

^§^Statistical significance determined using the one-way analysis of variance

^a^Differences at enrolment; ^b^differences at 6 months; ^c^differences at 12 months

*P−* *M−* mp negative and no clinical manifestation of malaria, *P+* *M−* mp positive and no clinical manifestation of malaria, *P+* *M+* malaria cases (febrile, mp positive+ at least one sign of malaria)

At baseline, 19.8 % of the participants were positive for intestinal helminths, which was significantly higher (p = 0.013) compared to the 10.2 % positive for intestinal helminths at 6 months. The majority of intestinal helminths observed were the soil-transmitted helminths *Ascaris*, *Trichuris* and hookworm. Other intestinal parasites (OIP) such as tapeworm (T), *Entameoba* (Enta), *Hymenolepis* (Hym) and *Enterobius* (Ente) were also detected in a few cases with some participants having multiple helminth infections. Among those infected with helminths at enrolment, 36.5 % were positive for *Ascaris lumbricoides* (Al), 34.9 % positive for *Trichuris trichuria* (Tt) 15.9 % positive for Hookworm (H), 6.3 % positive for *Ascaris* and *Trichuris*, 4.8 % positive for *Ascaris* and hookworm while 1.6 % were positive for *Ascaris*, *Trichuris* and hookworm. All helminth infections were light infections except for one moderate (according to WHO classification) case of *Ascaris* infection recorded at 6 months.

### Malaria morbidity during the 12 months longitudinal survey and relationship with baseline helminths status

**Fig. 1:**
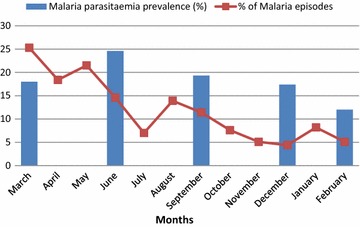
Malaria parasitaemia and malaria morbidity rates in a cohort of children over a 1 year period. Asymptomatic cases were only detected every 3 months while malaria episodes were detected throughout the year

Following the active and passive follow-up of participants for malaria, 32.5 % (116/357) of those who completed the longitudinal survey had at least one malaria episode and they constituted 71.2 % (116/163) of all those who visited a health establishment for consultation. The mean ± SEM number of malaria attacks per year was 1.44 ± 0.062 (range: 1–4) with the highest incidence of malaria attacks occuring during the rainy season months of March–October (Fig. 1). The mean ± SEM age of participants who had at least one episode of malaria was 4.06 ± 0.18 (range: 0.6–9 years) with 63.84 % (74/116) having only one attack while 36.2 % (42/116) had two or more attacks. The percentage of malaria parasitaemia positive cases increased from 18 % at enrolment (end of dry season) to 24.6 % at 3 months (rainy season) and decreased to 19.3 % at 6 months (rainy season), 17.4 % at 9 months (dry season) and 12 % at 1 year (dry season; Fig. 1).

Children less than 5 years old were significantly [OR = 0.47, 95 % CI (0.28–0.79), p = 0.004] less susceptible to having a malaria episode compared to children in the 5–10 years old age group in bivariate analysis after controlling for haemoglobin genotype. However, the study also showed that children <5 years old were significantly [OR = 2.34, 95 % CI (1.15–4.75), p = 0.019] exposed to more malaria attacks compared to children in the 5–10 years old age group (Table 2). Participants in the <5 years age group had significantly (p = 0.009) more malaria attacks compared to those in the 5–10 years age group (data not shown, Additional file 1). There was no difference in susceptibility to a malaria episode [OR = 1.39, 95 % CI (0.68–2.85), p = 0.360] between participants who were positive and those who were negative for helminths at baseline in multivariate analysis controlling for age and haemoglobin genotype. When the association to the frequency of malaria episodes was investigated in multivariate analysis after controlling for age and haemoglobin genotype, it was found that children positive for helminths had similar malaria episodes [OR = 0.49, 95 % CI (0.14–1.71), p = 0.266] compared to children who were negative for helminths at baseline (Table 2).

**Table 2:** Effect of age (years) and helminths status on malaria morbidity rates in a cohort of children during a 1 year longitudinal study

Parameter	Subclass	% (n)	Presence/absence of malaria episodes				Prevalence of malaria episodes				
			Unadjusted p value	OR	95 % CI	Adjusted p value	Unadjusted p value	OR	95 % CI		Adjusted p value
Age group (years)	<5	52.6 (199)	*0.005*	0.47	0.28–0.79	*0.004*	*0.019*	2.34	1.15–4.75		0.071
	5–10	45.5 (172)		REF				REF			
Helminths status	Positive	16.7 (63)	0.785	1.39	0.68–2.85	0.360	0.322	0.49	0.14–1.71		0.266
	Negative	67.5 (255)		REF				REF			
Haemoglobin genotype	AA	51.9 (196)	0.734	1.14	0.62–2.13	0.667	0.506	0.71	0.29–1.75		0.451
	AS	14 (53)		REF				REF			

Italic values represent statistically significant associations determined by binary logistic regression

### Anaemia in Cameroonian children living in Mutenegene

**Fig. 2:**
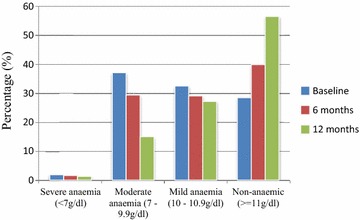
Classification and distribution of anaemia at three time points during a 1 year longitudinal study of children living in Mutengene, Cameroon

At enrolment 71.5 % of the participants were anaemic but decreased significantly (p < 0.001) to 60.1 % at 6 months and 43.5 % at 12 months (Fig. 2) and the proportion of participants in the different anaemia categories also decreased significantly (p < 0.001) across the three time points (Fig. 2). This was evident in a significant (p < 0.001) increase in haemoglobin levels [mean (g/dl) ± SEM] from 10.15 ± 0.06 at enrolment to 10.45 ± 0.08 at 6 months and finally to 11.01 ± 0.07 at 12 months (not shown). Haemoglobin levels (g/dl) of participants in the P+ M+ category were significantly lower compared to participants in the P+ M− category whose Hb levels (g/dl) were in turn significantly lower compared to participants in the P− M− category [p < 0.001 (Table 1)].

**Fig. 3:**
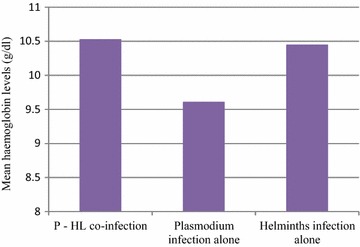
Mean haemoglobin levels (g/dl) by infection category

We found that children <5 years old were more susceptible to anaemia [OR = 2.24, 95 % CI (1.85–4.23), p = 0.013] compared to children in the 5–10 years age group after controlling for haemoglobin genotype, malaria parasitaemia status and helminths status in multivariate analysis (Table 3). Controlling for age and haemoglobin genotype, there was no difference (p = 0.092) in susceptibility to anaemia between children positive or negative for malaria parasitaemia. However, individually children with malaria episodes [OR = 4.45, 95 % CI (1.66–11.94), p = 0.003] as well as those with asymptomatic parasitaemia [OR = 2.41, 95 % CI (1.58–3.69) p < 0.001] were associated with susceptibility to anaemia compared to children who were malaria parasitaemia negative. Meanwhile children positive for helminths at baseline were protected [OR = 0.47, 95 % CI (0.22–0.97), p = 0.041] from anaemia compared to children who were negative for helminths. Co-infection with malaria parasites and helminths was however, not protective against anaemia controlling for age though the association (p = 0.310). Based on the presence and/or absence of *Plasmodium* or helminths infection or both, participants were grouped as: *Plasmodium*–helminths (P–HL) co-infection (2.9 %), *Plasmodium* alone (P) (13 %) and helminths alone (HL) (13.2 %). Considering children infected with *Plasmodium* alone as the reference and controlling for age, children infected with helminths alone were significantly associated with protection from anaemia [OR = 0.357, 95 % CI (0.141–0.901), p = 0.029] while those who were co-infected with both parasites were not protected from anaemia though the obvious association [OR = 0.291, 95 % CI (0.072–1.167), p = 0.081]. However, the mean ± SEM haemoglobin level (g/dl) of participants co-infected with *Plasmodium* and helminths (10.53 ± 0.62) was significantly (p = 0.006) higher compared to participants infected with *Plasmodium* (9.61 ± 0.19) or helminths (10.45 ± 0.17) alone (Fig. 3). In addition, a smaller proportion (2.6 %) of children co-infected with *Plasmodium* and helminths were anaemic compared to a higher (p = 0.017) proportion of children infected with *Plasmodium* alone (17.2 %) or helminths alone (13.7 %) at baseline (Additional file 2).

**Table 3:** Relationship between malaria, helminths, co-infection with anaemia in the study children population

Parameter	Subclass	% (n)	Anaemia			
			Unadjusted p value	OR	95 % CI	Adjusted p value
Age group (years)	<5	52.6 (199)	*<0.001*	2.24	1.85–4.23	*0.013*
	5–10	45.5 (172)		REF		
Helminths status	Positive	16.7 (63)	*0.021*	0.47	0.22–0.97	0*.041*
	Negative	67.5 (255)		REF		
Malaria parasitaemia status	Positive	17.2 (65)	0.839	2.00	0.89–4.48	0.092
	Negative	81 (306)		REF		
Manifestation	P+ M+	2.9 (29)	*0.008*	4.45	1.66–11.94	*0.003*
	P+ M−	12.7 (128)	*0.001*	2.42	1.81–3.08	*<0.001*
	P− M−	84.1 (844)		REF		
Haemoglobin genotype	AA	51.9 (196)	0.911	0.78	0.35–1.73	0.541
	AS	14 (53)		REF		
Co-infection Status	Yes	2.9 (11)	0.312	0.52	0.15–1.84	0.310
	No	26.2 (99)		REF		
Co-infection category	P − HL	2.9 (11)	0.093	0.29	0.07–1.17	0.081
	HL	13.2 (50)	*0.037*	0.36	0.14–0.90	*0.029*
	P	13 (49)		REF		

Italic values represent statistically significant associations assessed by binary logistic regression

*P−* *M−* mp negative and no clinical manifestation of malaria, *P+* *M−* mp positive and no clinical manifestation of malaria, *P+* *M+* malaria cases (febrile, mp positive + at least one sign of malaria), *P* *−* *HL* *Plasmodium*–helminths co-infection, *P* *Plasmodium* alone, *HL* helminths alone

Malaria parasitaemia density/µl was found to correlate negatively with haemoglobin levels [(g/dl) r = −0.37; p = 0.002], hookworm density [(eggs/g) r = −1.0; p < 0.001] and positively with *Ascaris* density (r = 0.67; p = 0.072), *Trichuris* density (r = 0.26; p = 0.742) and the density of other intestinal parasites (r = 0.95; p = 0.051) though correlation with the latter group was insignificant. Haemoglobin levels (g/dl) correlated positively with *Ascaris* density (r = 0.13; p = 0.488) and negatively with *Trichuris* density (r = −0.031; p = 0.876) as well as hookworm density (r = −0.18; p = 0.544) though insignificantly. However, haemoglobin levels (g/dl) correlated negatively (r = −0.753; p = 0.031) with the density of other intestinal parasites.

## Discussion

There is published data on malaria and helminths co-infection in the south west region of Cameroon [4, 11, 23] but there is none assessing the impact of malaria and helminths co-infection on the incidence of malaria episodes. This longitudinal survey of a cohort of 357 Cameroonian children living in Mutengene assessed their clinical, parasitological (*Plasmodium* and intestinal helminths) and anaemia status and evaluated the relationship between malaria parasitaemia, malaria episodes, anaemia and helminths status. The malaria parasitaemia prevalence observed in this study was lower compared to that reported by Nkuo-Akenji et al. [11] and Achidi et al. [4] although these studies were conducted in different towns of the South West Region of Cameroon. This can be explained by the scaling up of health education and the implementation of malaria control strategies in recent years especially the mass distribution of free insecticide treated bed nets in the study area. Majority of the children who were malaria parasitaemic during the study were 5–10 years old but a greater proportion of those who had asymptomatic parasitaemia belonged to this age group compared to healthy malaria parasitaemia negative children and those who had episodes of malaria. The mean age of children who had at least one malaria episode was 4.06 years and children less than 5 years old had more attacks compared to those who were 5–10 years old. Though older children (5–10 years) were more susceptible to *Plasmodium* infection, children less than 5 years old were exposed to more malaria episodes during the 1 year study period. This observation is in accordance with longstanding published reports that children below 5 years old are more susceptible to malaria. Malaria parasitaemia was predominantly associated with clinical manifestation as children who had malaria episodes had significantly higher malaria parasitaemia densities compared to those who were malaria parasitaemia positive without clinical symptoms. The highest incidence of malaria was observed in the month of March, which corresponds to the transition from the dry season to the rainy season (moderate rains), the incidence reduced slightly in the month of June (peak rainy season) and this coincided with the highest malaria prevalence recorded during the study period.

Although the aetiology of anaemia is complex and multi-factorial, parasitic diseases, including *P. falciparum* and helminth infections, have long been recognized as major contributors to anaemia in endemic countries. A very high prevalence of anaemia was recorded at baseline (71.5 %) in contrast to the lower prevalences of 30.8, 11.9 and 10.9 % reported by Nkuo-Akenji et al. [11], Achidi et al. [4] and Alemu et al. [13], respectively, but similar to the prevalence reported by Makoge et al. [23]. Children below the age of 5 years were more susceptible to anaemia in accordance with facts that this age group is most vulnerable to malaria and its consequences. Malarial anaemia is more typically associated with the acute clinical state, but there is evidence to suggest that asymptomatic parasitaemia may contribute substantially to anaemia in endemic regions [10]. A greater proportion of participants who were malaria parasitaemia positive were anaemic throughout the study although only a small proportion of the children who were anaemic were actually malaria parasitaemia positive. This implies that despite the fact that malaria parasitaemia was an important cause of anaemia there were obviously other significant contributing factors. Nevertheless, malaria parasitaemia was the major contributor of anaemia as haemoglobin levels of children who experienced malaria episodes were lower compared to those with asymptomatic parasitaemia and those who were malaria parasitaemia negative. In addition, children with malaria episodes as well as those with asymptomatic parasitaemia were associated with susceptibility to anaemia compared to children who were malaria parasitaemia negative.

The baseline prevalence of intestinal helminths decreased from 19.8 to 10.2 % 6 months later probably due to the general deworming of all participants at enrolment and emphasis on the need for regular deworming. However it is worth mentioning that single stool specimen examination was employed in this case thus the possibility of reduced sensitivity. The helminths detected were almost entirely composed of the soil transmitted helminths *Ascaris lumbricoides*, *Trichuris trichuria* and the hookworms. This prevalence was comparable to that observed by Achidi et al. [4] but lower when compared to that reported by Nkuo-Akenji et al. [11] and Alemu et al. [13]. Co-infecting parasites may interact either positively or negatively in the receptive hosts [6, 10] through a range of mechanisms including resource competition, immune-mediated interactions and direct interference [7, 8]. Among the children positive for helminths at baseline, a greater proportion (58.7 %) were anaemic confirming previous reports that helminth infections are significant contributors to anaemia (Additional file 3). Human populations of helminths and malaria co-infection have shown contradictory results for the course of the malaria infection and disease possibly depending on the type of helminth studied, the intensity of the helminth infection and the age of the study population [5]. A smaller proportion of children co-infected with *Plasmodium* and helminths were anaemic compared to a higher proportion of children infected with *Plasmodium* alone or helminths alone and this agrees with what was observed by Nkuo-Akenji et al. [11], but is in contrast with reports by Degarege et al. [6]. Likewise haemoglobin levels of children who were co-infected were higher compared to children infected with either *Plasmodium* or helminths alone. Co-infection with both parasites was protective against anaemia whereas children infected with *Plasmodium* alone were more susceptible to anaemia. This is probably because the outcome of *Plasmodium*–helminth interactions is beneficial to the host in that the individual negative effect of both infections on the host cancel out. The successful resolution of *Plasmodium* infection requires a coordinated succession from a T-helper cell type 1 (Th1) to a Th2 type response, and anything that upsets the timing or balance of this process can lead to chronic or severe infection [5]. Infection with helminths has a profound effect on the immune system resulting in polarization towards Th2, characterized by high levels of cytokines such as interleukin-4 (IL-4), IL-5, IL-13 and high serum levels of immunoglobulin E [2]. The long-term survival of the helminths is thought to be facilitated by the induction of immuno-regulatory mechanisms that include the induction of regulatory T cells [2, 7] and modulation of cells of the innate immune system, such as macrophages and dendritic cells, which result in an anti-inflammatory environment, characterized by increased levels of IL-10 and TGF-β [1] and possibly limiting pro-inflammatory mediated harmful consequences such as anaemia.

The negative correlation of malaria parasitaemia with haemoglobin levels and hookworm consolidates the fact that malaria parasitaemia was an important contributor of anemia while hookworms and *Plasmodium* seemed to interact negatively. Actually no co-infection was recorded involving hookworm infection in contrast to Naing et al. [8] who reported a positive association between hookworms and asymptomatic malaria. Though insignificant the observed positive correlation between malaria parasitaemia density and the densities of *Ascaris*, *Trichuris* and other intestinal parasites seem to imply that these two groups of parasites interact positively. However, the malaria parasitaemia densities of children positive for *Trichuris* was higher compared to children positive for *Ascaris* (data not shown, Additional file 4) in agreement with reports by Mulu et al. [9] that *Trichuris* infection was associated with increased malaria parasitaemia. *Ascaris* infection did not appear to have any effect on haemoglobin levels while both Trichuris and hookworm infections seemed to have a negative effect and infection with the other intestinal parasites clearly had a negative effect on haemoglobin levels. In view of the variation in routes of entry to the host and the different clinical outcomes, the interaction of these helminths with malaria parasites is expected to be different from one another [8].

No significant association of susceptibility to malaria during the study was observed between children positive or negative for helminths. Previous association of helminth infection with an increased risk for malaria incidence has been confirmed in two other studies. The first study reported a positive association between intestinal helminths and infection with *P. falciparum* in adults in Thailand, whereas the second study on mothers and children in Zaire described a positive association between infection with *Ascaris* and the occurrence of *P. falciparum* as reviewed by Hartgers and Yazdanbakhsh, [1]. However, though children positive for helminths seemed to be exposed to the same number of malaria episodes as children who were negative for helminthes, results from a cohort of people on the Thai-Myanmar border have shown that helminth-infected patients had a twofold increase of falciparum malaria incidence (fever + malaria parasites on the blood smear) [12]. In a review, Nacher [24] reports increased gametocyte carriage in a human study and increased malaria transmission in an animal model of helminth-malaria co-infection. In the absence of disease, patients co-infected with worms and malaria may represent a hub of malaria transmission [24]. A recent meta-analysis of mouse co-infection studies suggests that depending on the existing immune interaction between a given host and malaria parasites, addition of a helminth co-infection may have contrasting effects on malarial disease: co-infection increased mortality and peak parasitaemia in ordinarily resolving *Plasmodium* infections, but had a far less effect on lethal *Plasmodium* infections and even tended to delay death in cerebral malaria models [7].

## Conclusion

Children less than 5 years old were more susceptible to malaria and anaemia. The prevalence of anaemia in this community was quite high and was largely due to malaria parasitaemia and to a smaller extent to helminth infection. In addition, nutritional factors probably also contributed especially to the anaemia observed at baseline. Malaria and helminths co-infection was rather beneficial to the children as this was associated with protection from anaemia. Studies assessing the effect of helminth infections on the course of malaria are very important in target malaria vaccine trial communities. Well-structured longitudinal studies investigating the underlying immune mechanisms involved in malaria helminths co-infection are very important in this regard. Our results also indicate that employing the proper intervention in a combined malaria–helminth approach will yield timely results in endemic communities (Additional file 5).

## Additional files

10.1186/s12936-016-1111-2 Number of malaria attacks by age group and gender. Proportion of participants with one attack vs multiple attacks by age group and gender.
10.1186/s12936-016-1111-2 Frequency distribution of severity of anaemia by infection category. Proportion of participants with varying severity of anaemia by infection category (P-HL co-infection, *Plasmodium* only, helminths only).
10.1186/s12936-016-1111-2 Frequency distribution of age group, sex and anaemia status by helminth status. Proportion of participants who were helminth positive/negative by age group, gender and anaemia status at three time points.
10.1186/s12936-016-1111-2 Geometric mean malaria parasitaemia density/ul of participants positive for *Ascaris* and *Trichuris.* Comparison of malaria parasitaemia density/ul between participants positive for *Ascaris* and participants positive for *Trichuris* at baseline.
10.1186/s12936-016-1111-2 Geometric mean haemoglobin levels and malaria parasitaemia density of children at three time points. Comparison of mean haemoglobin level and mean malaria parasitaemia density/ul at three time points.

## References

[1] Hartgers FC, Yazdanbakhsh M (2006). Co-infection of helminths and malaria: modulation of the immune responses to malaria. Parasite Immunol.

[2] Mwangi TW, Bethony J, Brooker S (2006). Malaria and helminth interactions in humans: an epidemiological viewpoint. Ann Trop Med Parasitol.

[3] Brooker S, Akhwale W, Pullan R, Estambale B, Clarke SE, Snow RW (2007). Epidemiology of *Plasmodium*–helminth co-Infection in Africa: populations at risk, potential impact on anemia, and prospects for combining control. Am J Trop Med Hyg.

[4] Achidi EA, Apinjoh TO, Mbunwe E, Besingi R, Yafi CN, Awah NC (2008). Febrile status, malaria parasitaemia and gastrointestinal helminthiasis in school children resident at different altitudes. Ann Trop Med Parasitol.

[5] Hartgers FC, Obeng BB, Boakye D, Yazdanbakhsh M (2008). Immune responses during helminth-malaria co-infection: a pilot study in Ghanaian school children. Parasitology.

[6] Degarege A, Animut A, Legesse M, Erko B (2010). Malariaand helminth co-infections in outpatients of Alaba Kulito Health Center, southern Ethiopia: a cross sectional study. BMC Res Notes.

[7] Knowles SCL (2011). The effect of helminth co-infection on malaria in mice: A meta-analysis. Int J Parasitol.

[8] Naing C, Whittaker MA, Nyunt-Wai V, Reid SA, Wong SF, Mak JW (2013). Malaria and soil-transmitted intestinal helminth co-infection and its effect on anemia: a meta-analysis. Trans R Soc Trop Med Hyg.

[9] Mulu A, Legesse M, Erko B, Belyhun Y, Nugussie D, Shimelis T (2013). Epidemiological and clinical correlates of malaria-helminth co-infections in southern Ethiopia. Malar J.

[10] Pullan R, Brooker S (2008). The health impact of polyparasitism in humans: are we under-estimating the burden of parasitic diseases?. Parasitology.

[11] Nkuo-Akenji TK, Chi PC, Cho JF, Ndamukong KKJ, Sumbele I (2006). Malaria and helminth co-infection in children living in a malaria endemic setting of mount cameroon and predictors of anemia. J Parasitol.

[12] Nacher M (2008). Worms and malaria: blind men feeling the elephant?. Parasitology.

[13] Alemu A, Shiferaw Y, Ambachew A, Hamid H (2012). Malaria helminth co-infections and their contribution for aneamia in febrile patients attending Azzezo health center, Gondar, northwest Ethiopia: a cross sectional study. Asian Pacific J Trop Med.

[14] Wanji S, Tanke T, Atanga SN, Ajonina C, Nicholas T, Fontenille D (2003). Anophheles species of the Mount Cameroon region; biting habits, feeding behaviour and entomological inoculation rates. Trop Med Int Health.

[15] Achidi EA, Anchang JK, Minang TJ, Boyo MA, Sinju CM, Mokube J (2005). Studies on *Plasmodium falciparum* isotypic antibodies and numbers of IL-4 and IFN-γ secreting cells in paired maternal cord blood from south west Cameroon. Int J Infect Dis.

[16] Cameroon Development Corporation (CDC). Meteorological service, Esuke Station (2011). http://www.cdc-cameroon.com/subpages/. Accessed 20 June 2014.

[17] Titanji VPK, Tamu VD, Akenji TKN, Joutchop AS (2002). Immunoglobulin G and subclass responses to *Plasmodium falciparum* antigens: a study in highly exposed cameroonians. Clin Chem Lab Med.

[18] Nkuo-Akenji TK, Ntonifor NN, Ching JK, Kimbi HK, Ndamukong KN (2004). Evaluating a malaria intervention strategy using knowledge, practices and coverage surveys in rural Bolifamba, southwest Cameroon. Trans R Soc Trop Med Hyg.

[19] Tanga MC, Ngundu WI, Tchouassi PD (2011). Daily survival and human blood index of major malaria vectors associated with oil palm cultivation in Cameroon and their role in malaria transmission. Trop Med Int Health.

[20] Bigoga JD, Manga L, Titanji VPK, Coetzee M, Leke RGF (2007). Malaria vectors and transmission dynamics in coastal south-west Cameroon. Malar J.

[21] World Health Organization. http://www.who.int/vmnis/indicators/haemoglobin. Haemoglobin concentrations for the diagnosis of anaemia and assessment of severity. 2011. Accessed 24 May 2013.

[22] Ash LR, Orihel TC (1991). A guide to laboratory procedures and identification.

[23] Makoge VD, Mbah GA, Nkengazong L, Sahfe NE, Moyou RS (2012). Falciparum malaria, helminth infection, and anaemia in asymptomatic pupils in four villages in Cameroon. Eur J Zool Res.

[24] Nacher M (2012). Helminth-infected patients with malaria: a low profile transmission hub?. Malar J.

