# Supplementary material for: Malaria, helminths, co-infection and anaemia in a cohort of children from Mutengene, south western Cameroon
Source: Malar J. 2016 Feb 6;15:69. doi: 10.1186/s12936-016-1111-2 (PMC4744422; doi:10.1186/s12936-016-1111-2)
Supplement: Supplementary file 1 — 10.1186/s12936-016-1111-2 Number of malaria attacks by age group and gender. Proportion of participants with one attack vs multiple attacks by age group and gender. [file 12936_2016_1111_MOESM1_ESM.docx]

Additional file 1: Number of malaria attacks by age group and gender

| **Number of malaria attacks** | **Age group** | | **Gender** | |
| --- | --- | --- | --- | --- |
|  | < 5years old (%) | 5-10years old (%) | Male (%) | Female (%) |
| **One attack** | 38 (51.4) | 36 (48.6) | 41 (50.6) | 40 (49.4) |
| **2 or more attacks** | 32 (76.2) | 10 (23.8) | 26 (61.9) | 16 (38.1) |
| **Total** | 70 (60.3) | 46 (39.7) | 67 (54.5) | 56 (45.5) |
| **P value** | **χ^2^ = 6.91** **p = 0.009** | | χ^2^ =1.42 p = 0.233 | |

Proportion of participants with one attack vs multiple attacks by age group and gender
