# Supplementary material for: Malaria, helminths, co-infection and anaemia in a cohort of children from Mutengene, south western Cameroon
Source: Malar J. 2016 Feb 6;15:69. doi: 10.1186/s12936-016-1111-2 (PMC4744422; doi:10.1186/s12936-016-1111-2)
Supplement: Supplementary file 2 — 10.1186/s12936-016-1111-2 Frequency distribution of severity of anaemia by infection category. Proportion of participants with varying severity of anaemia by infection category (P-HL co-infection, Plasmodium only, helminths only). [file 12936_2016_1111_MOESM2_ESM.docx]

**Additional file 2**: Frequency distribution of severity of anaemia by infection category

| **Sampling period** | **Infection category** | **Severity of anaemia** | | | **Total** | **Level of significance** |
| --- | --- | --- | --- | --- | --- | --- |
|  |  | **Severe anaemic (Hb<7g/dl)** | **Moderate anaemia (Hb: 7-9.9g/dl)** | **Mild anaemia (Hb: 10-10.9g/dl)** |  |  |
| **Enrolment** | *P - HL co-infection (%) | 1 (16.7) | 1 (0.8) | 4 (4) | 6(2.6) | **χ^2^ =15.51**  **p = 0.017** |
|  | *Plasmodium* only (%) | 3 (50) | 24 (20) | 12 (11.9) | 39 (17.2) |  |
|  | Helminthes only (%) | 0 (0) | 14 (11.7) | 17 (16.8) | 31 (13.7) |  |
|  | No infection (%) | 2 (33.3) | 81 (67.5) | 68 (67.3) | 151 (66.5) |  |
|  | Total | 6 (100) | 120 (100) | 101 (100) | 227 (100) |  |
| **6 months** | P - HL co-infection (%) | 0 (0) | 0 (0) | 1 (2.6) | 1 (1.1) | χ^2^ = 5.16  p = 0.271 |
|  | *Plasmodium* only (%) | 1 (100) | 12 (21.8) | 9 (23.1) | 22 (23.2) |  |
|  | Helminthes only (%) | 0 (0) | 5 (9.1) | 4 (10.3) | 9 (9.5) |  |
|  | No infection (%) | 0 (0) | 38 (69.1) | 25 (64.1) | 63 (66.3) |  |
|  | total | 1 (100) | 55 (100) | 39 (100) | 95 (100) |  |

Proportion of participants with varying severity of anaemia by infection category. *P – HL co-infection: *Plasmodium*-helminths co-infection
