# Supplementary material for: Malaria, helminths, co-infection and anaemia in a cohort of children from Mutengene, south western Cameroon
Source: Malar J. 2016 Feb 6;15:69. doi: 10.1186/s12936-016-1111-2 (PMC4744422; doi:10.1186/s12936-016-1111-2)
Supplement: Supplementary file 3 — 10.1186/s12936-016-1111-2 Frequency distribution of age group, sex and anaemia status by helminth status. Proportion of participants who were helminth positive/negative by age group, gender and anaemia status at three time points. [file 12936_2016_1111_MOESM3_ESM.docx]

**Additional file 3**: Frequency distribution of age group, gender and anaemia status by helminth status

| **Sampling period** | **Helminth Status** | **Age group**  **(%)** | | **Gender**  **(%)** | | **Anaemia status**  **(%)** | |
| --- | --- | --- | --- | --- | --- | --- | --- |
|  |  | **< 5 years** | **5 - 10 years** | **Male** | **Female** | **Anaemic** | **Non-anaemic** |
| **Enrolment** | **Positive (%)** | 32 (50.8) | 31 (49.2) | 32 (50.8) | 31 (49.2) | 37 (58.7) | 26 (41.3) |
|  | **Negative (%)** | 133 (52.2) | 122 (47.8) | 137 (53.5) | 119 (46.5) | 188 (73.7) | 67 (26.3) |
|  | **Total** | 165 | 153 | 169 | 150 | 225 | 93 |
|  | **Level of significance** | χ^2^ = 0.04 p = 0.846 | | χ^2^ = 0.15 p = 0.698 | | **χ^2^ =5.49 p = 0.019** | |
| **6 months** | **Positive (%)** | 13 (76.5) | 4 (23.5) | 9 (52.9) | 8 (47.1) | 10 (58.8) | 7 (41.2) |
|  | **Negative (%)** | 63 (44.7) | 78 (55.3) | 72 (51.1) | 69 (48.9) | 85 (60.7) | 55 (39.3) |
|  | **Total** | 76 | 82 | 81 | 77 | 95 | 62 |
|  | **Level of significance** | **χ^2^ = 6.14 p = 0.013** | | χ^2^ = 0.02 p = 0.884 | | χ^2^ = 0.023 p = 0.88 | |

Proportion of participants who were helminth positive/negative by age group, gender and anaemia status at three time points
