# Supplementary material for: Malaria, helminths, co-infection and anaemia in a cohort of children from Mutengene, south western Cameroon
Source: Malar J. 2016 Feb 6;15:69. doi: 10.1186/s12936-016-1111-2 (PMC4744422; doi:10.1186/s12936-016-1111-2)
Supplement: Supplementary file 4 — 10.1186/s12936-016-1111-2 Geometric mean malaria parasitaemia density/ul of participants positive for Ascaris and Trichuris. Comparison of malaria parasitaemia density/ul between participants positive for Ascaris and participants positive for Trichuris at baseline. [file 12936_2016_1111_MOESM4_ESM.docx]

Additional file 4: Baseline geometric mean malaria parasitaemia density/ul of participants positive for *Ascaris* and *Trichuris*

| **Helminth species** | **N** | **Malaria parasitaemia density /ul ± SEM** | **Level of significance** |
| --- | --- | --- | --- |
| **Trichuris** | 3 | 3.96 ± 0.33 | t =2.80 **p = 0.038** |
| **Ascaris** | 4 | 2.75 ± 0.28 |  |

Comparison of malaria parasitaemia density/ul between participants positive for *Ascaris* and participants positive for *Trichuris* at baseline
