# Supplementary material for: Malaria, helminths, co-infection and anaemia in a cohort of children from Mutengene, south western Cameroon
Source: Malar J. 2016 Feb 6;15:69. doi: 10.1186/s12936-016-1111-2 (PMC4744422; doi:10.1186/s12936-016-1111-2)
Supplement: Supplementary file 5 — 10.1186/s12936-016-1111-2 Geometric mean haemoglobin levels and malaria parasitaemia density of children at three time points. Comparison of mean haemoglobin level and mean malaria parasitaemia density/ul at three time points. [file 12936_2016_1111_MOESM5_ESM.docx]

**Additional file 5**: Geometric mean haemoglobin levels and malaria parasitaemia density/ul of children at three time points

| **Sampling period** | **Haemoglobin level (g/dl)** | **Mp density (/µl)** |
| --- | --- | --- |
| **Enrolment** | 10.15 ± 0.06 | 3.09 ± 0.01 |
| **6 months** | 10.45 ± 0.08 | 2.97 ± 0.08 |
| **12 months** | 11.01 ± 0.07 | 2.80 ± 0.16 |
| **P value** | **<0.001** | 0.219 |

Comparison of mean haemoglobin level and mean malaria parasitaemia density/ul at three time points
